# Objectively measured physical activity and sedentary behaviour in children with bronchiectasis: a cross-sectional study

**DOI:** 10.1186/s12890-018-0772-8

**Published:** 2019-01-08

**Authors:** Barbara Joschtel, Sjaan R. Gomersall, Sean Tweedy, Helen Petsky, Anne B. Chang, Stewart G. Trost

**Affiliations:** 1 School of Human Movement and Nutrition Sciences, The University of Queensland, Brisbane, Australia; 2 School of Health & Rehabilitation Sciences, The University of Queensland, Brisbane, Australia; 3 School of Nursing and Midwifery, Griffith University, Brisbane, Australia; 4 Child Health Division, Menzies School of Health Research, Charles Darwin University, Darwin, Australia; 5 Institute of Health and Biomedical Innovation, Centre for Children’s Health Research, Queensland University of Technology, Brisbane, Australia; 6 Department of Respiratory Medicine, Queensland Children’s Hospital, Children’s Health Queensland, Brisbane, Australia; 7 QLD Centre for Children’s Health Research (CCHR), Level 6, 62 Graham Street, South Brisbane, QLD 4101 Australia

**Keywords:** Bronchiectasis, Children, Physical activity, Accelerometer, Physical activity guidelines, Sedentary behavior, Steps

## Abstract

**Background:**

Bronchiectasis is a major contributor to respiratory morbidity and health care utilization in children and youth. Current treatment guidelines for bronchiectasis recommend participation in regular physical activity (PA) to improve aerobic fitness and quality of life (QoL). However, no previous study has assessed physical activity and sedentary behavior in this patient group, and the extent to which children with bronchiectasis meet guidelines for PA is unknown. In the absence of such data, we objectively measured the PA of children with bronchiectasis and compared them to current guidelines.

**Methods:**

Forty-six children with bronchiectasis between 4 to 14 years (mean age 7.5 ± 2.6 years) were recruited from the Queensland Children’s Hospital, Brisbane. Daily time in sedentary, light, and moderate-to-vigorous PA (MVPA) was measured objectively over 7 days using the ActiGraph GT3X+ accelerometer and compared their values to current guidelines (minimum 60 min of MVPA daily). Compliance with the daily guideline and average daily steps counts were compared to normative data from two population–based health surveys of healthy children.

**Results:**

We had complete measurements from 36 children. On average, they accumulated 48.6 min of MVPA daily and were sedentary for ~ 7 h/day. There was no statistical difference in these values between sexes or weekdays vs. weekends. Only 2 (5.6%) children met the 60-min daily MVPA recommendation compared to 42.1% of healthy children. Children with bronchiectasis accumulated 8229 steps/day (boys: 8422 ± SD 473, girls: 8037 ± 594), well below the recommended 12,000 steps/day. In comparison, daily step counts in healthy children ranged from 11,500–14,500 steps/day.

**Conclusion:**

Children with bronchiectasis are insufficiently active for health benefit and would substantially benefit from programs to promote PA and reduce sedentary behavior.

## Introduction

Bronchiectasis is an increasing major contributor to respiratory morbidity and healthcare utilization in children and youth [1–5]. Yet, it is one of the most neglected lung diseases [6]. It is the end point of the chronic suppurative lung disease (CSLD) continuum and is described as abnormal irreversible dilatation of the airways, related with airway infection and inflammation [1, 2]. The occurrence of exacerbations or flare ups (increased wetness and severity of cough, breathlessness, chest pain, and/or wheeze), leads to frequent hospitalization and further decline in lung function [4, 5]. Prevalence data in children are scarce; however, a recent review of the epidemiology of CLSD estimated the prevalence of bronchiectasis to range from 0.2 to 15 cases per 100,000 [3]. Bronchiectasis is particularly prevalent among socially disadvantaged populations, such as Indigenous communities of Australia, New Zealand, Alaska and Canada [2, 3]. A study of Central Australian Indigenous children reported the prevalence as high as one in every 68 Indigenous children [2].

A child’s participation in active play and exercise is one of the most important indicators of his or her overall health and well-being. Among healthy children, physical activity is associated with a range of health benefits such as improved aerobic fitness, musculoskeletal health, and psychosocial health, while inversely related to a number of cardio-metabolic risk factors including obesity, elevated blood lipids, glucose intolerance and hypertension [7–9]. On the basis of this evidence, government agencies and global health organizations recommend that children and0020adolescents participate in at least 60-min of moderate-to-vigorous intensity physical activity (MVPA) every day [10–13].

Current guidelines for the treatment and management of bronchiectasis recommend participation in regular physical activity as a means of improving aerobic fitness and health related quality of life (QoL) [4]. However, no previous study has assessed physical activity in this patient group and the extent to which children with bronchiectasis meet guidelines for physical activity is unknown. In addition, there is no information about levels of sedentary behavior in this patient group. Emerging evidence suggests that sedentary behavior, characterized by prolonged bouts of sitting, is associated with short- and long-term health risks, independent of the effects of physical activity [14]. To date, studies assessing physical activity and/or sedentary behavior in children with other respiratory conditions such as asthma and cystic fibrosis have produced conflicting results. While some studies suggest that children with respiratory disease are less active than the general child population [15–21], other studies report no differences [22–25]. The discrepancy in findings may be attributable, at least in part, to the use of self-report measures of physical activity with limited validity and/or differences in accelerometer data reduction methodology [26].

With growing recognition that regular physical activity plays a key role in promoting QoL and preventing disabling secondary conditions such as obesity, depression, and anxiety [7–9], it is imperative to learn more about the physical activity and sedentary behaviors of children with bronchiectasis. Therefore, in children with bronchiectasis, we aimed to: 1) objectively measure their physical activity and sedentary behaviors and; 2) examine their compliance with the current guideline of a minimum of 60 min of MVPA daily.

## Methods

### Participants and setting

Children meeting the following inclusion criteria were eligible to participate in the study: diagnosed with bronchiectasis, aged between 4 and 13 years and willing to wear an accelerometer. Bronchiectasis was diagnosed according to guidelines published by the Thoracic Society of Australia and New Zealand [4]. Exclusion criteria were as follows: unstable medical condition; unstable emotional or behavioral status; recent musculoskeletal injuries e.g., sprains, fracture, muscle strains. Participants were recruited through the Respiratory and Sleep Department at the Queensland Children’s Hospital, Brisbane Australia. Clinicians within the department were provided with a detailed description of the study, along with the inclusion and exclusion criteria, and asked to identify children who met the criteria and would be interested in participating in a study addressing movement competence and physical activity. Parents of potential participants were subsequently contacted by a member of the research team who formally assessed study eligibility and provided details about the study. In total, 55 patients were referred into the study. After discussing the study, parents with children who were eligible and interested in participating provided written informed consent. Children aged between 7 and 13 years also provided written assent. Ethical approval for this study was received from the Human Research Ethics committee at the Children’s Health Queensland Hospital and Health Service (HREC/14/QRCH/136) and the Human Research Ethics committee at the University of Queensland (2014001176).

### Measures

#### Parent questionnaire

Parents completed a questionnaire asking about the child’s type (e.g. wet) and length of cough, use of current medications, frequency of doctor visits in the past 12 months, and frequency and severity of exacerbations in the last 12 months. Additional items measured family structure, parental age, parental smoking status, parental occupation, and household income.

#### Measurement of Physical Activity

Physical activity was objectively measured using the ActiGraph GT3X+ accelerometer (ActiGraph Corporation, Pensacola, Fl, USA). Participants were asked to wear the accelerometer on the right hip for seven consecutive days during the waking hours, except for bathing and water based activities. Participants completed a brief log to record their sleep and wake times and report any time periods the monitor was removed for water-based activities or other reasons. The physical activity assessments were completed on a rolling basis over a 12-month period, beginning in March 2015 and ending in February 2017.

Accelerometers were initialized and downloaded using the ActiLife software (Version 6.13.3, ActiGraph, Pensacola, Fl, USA). After completing the 7-d monitoring protocol, the stored data were uploaded to a customized Visual Basic EXCEL macro to calculate daily wear time and time spent in sedentary (SED), light-intensity physical activity (LPA), moderate-intensity physical activity (MPA), vigorous-intensity physical activity (VPA), and moderate- to vigorous-intensity physical activity (MVPA). Activity counts recorded by the ActiGraph were classified into the aforementioned intensity categories using the cut-points developed by Evenson and colleagues [27] (SED/LPA ≥ 25 counts per 15 s, LPA/MVPA ≥574 counts per 15 s), which have been shown to be the most accurate of all currently available ActiGraph cut points for youth [28]. Non-wear time was defined as intervals with at least 60 consecutive minutes of zero counts, with allowance for up to 2 min under the count threshold for sedentary activity [29]. Daily wear time was calculated by subtracting non-wear time from the total monitoring time for the day. A day was considered valid if daily wear time exceeded 480 min. Participants were included in the analyses if they had 4 or more valid monitoring days including at least 1 weekend day [18]. Participants were classified as meeting the physical activity guideline if they accumulated ≥60 min of MVPA on each valid monitoring day.

#### Normative data comparisons

To benchmark the physical activity levels of children with bronchiectasis with healthy children residing in the same geographical region, the percentage of children meeting the 60-min MVPA guideline and average daily steps counts were compared to estimates from two population representative samples of Queensland children – the Queensland Preventive Health Survey [30] and the Healthy Kids Queensland Survey [31].

The Queensland Preventive Health Survey is a telephone survey conducted annually by the Queensland Government Department of Health [30]. Data are collected from a random sample of Queensland adults and children. One adult is selected from a contacted household to complete the survey. Parents answered questions about their child’s health. For their child’s physical activity, parents were asked following question: “over the past 7 days, on how many days was your child physically active for a total of 60 minutes or more per day?” A child was classified as meeting guidelines for physical activity if they had been active for ≥60 min every day over the last 7 days. In the 2015–2016 survey, physical activity data were collected on 5025 children between the ages of 5–17 years.

The Healthy Kids Queensland Survey [31] was commissioned by the Queensland Government Department of Health to obtain data to develop and implement effective policies and program to improve dietary and physical activity in Queensland children. A total of 3691 children in Grades 1, 5, and 10 from 112 randomly selected public and private schools participated in the survey. Participants wore a pedometer for five consecutive days, including weekdays and weekend days.

### Statistical analysis

Descriptive characteristics for the physical activity variables were described using means, standard deviations, 95% confidence intervals (CI’s) and frequencies. Sex differences in the physical activity variables were evaluated for statistical significance using one-way ANCOVA, with age and accelerometer wear time included as covariates. Weekday versus weekend differences in the physical activity variables were evaluated for statistical significance using a two-way (gender x time) ANCOVA, with age and accelerometer time included as covariates. All statistical analyses were performed using SPSS software version 26 (IBM Corp, Armonk, New York). Statistical significance was set at 2-sided alpha level of 0.05.

## Results

Of the 55 patients referred to the study, 46 children (83.6%) participated in the study. Of this number, 36 had the minimum requirement of 4 valid monitoring days, including 1 valid weekend day. The mean ± SD number of valid monitoring days was 5.9 ± 1.3 days, with an average daily wear time of 728.2 ± 114.6 min. Descriptive characteristics for the total sample and final monitoring sample are reported in Table 1. The exclusion of these ten participants had relatively little impact on the sample characteristics; however, the final analytic sample had a smaller percentage of children with ≥20 doctor visits in the previous year and a smaller percentage of households with an annual income of < AUD $25,000. More than half of the participants were currently taking medications including inhaled or oral steroids, bronchodilators or antibiotics and the majority of the children (41.7%) reported 5 to 9 doctor visits during the last 12 months. About 26% of the families had low family income below the Australian poverty line (single adult income of < $426.30/week) [32].

**Table 1:** Descriptive statistics of all recruited children (*n* = 46) and children (*N* = 36) with 4 or more days of physical activity data, including 1 weekend day

	*N* = 46	*N* = 36
Age	7.5 ± 2.6 years	7.7 ± 2.7 years
Male	63% (48.6–75.5)	61.1% (44.9–75.2)
Currently on Medication	60.9% (46.5–73.6)	63.9% (47.6–77.5)
Inhaled steroids	21.7% (12.3–35.6)	22.2% (11.7–38.1)
Bronchodilators	23.9% (13.9–37.9)	19.4% (9.8–35.0)
Oral steroids	4.3% (1.2–14.5)	2.8% (0.5–14.2)
Antibiotics	41.3% (28.3–55.7)	38.9% (24.8–55.1)
Number of doctors’ visits in the last 12 months		
< 5 times	26.1% (15.6–40.3)	30.6% (2.9–21.8)
5–9 times	39.1% (26.4–53.5)	41.7% (27.4–57.8)
10–20 times	23.9% (13.9–37.9)	22.2% (11.7–38.1)
> 20 times	10.9% (4.7–23.0)	5.6% (1.5–18.2)
Single parent household	17.4% (9.1–30.7)	19.5% (9.8–35.0)
Average household income		
< 25,000 AUD	18.2% (10.7–33.2)	11.8% (4.4–25.3)
26,000–50,000 AUD	10.9% (4.7–23.0)	16.7% (7.9–31.9)
51,000–75,000 AUD	2.2% (0.4–11.3)	0%
> 76,000 AUD	67.4% (53.0–79.1)	72.2% (56.0–84.2)
Families with a smoker	21.7% (12.3–35.6)	25.0% (13.8–41.1)

Table 2 displays means and 95% CI’s for the physical activity variables adjusted for age and accelerometer wear time. On average, participants accumulated 48.6 min of MVPA and 260.9 min of LPA, daily. Participants were sedentary for an average of 418.7 min, or approximately 7 h per day. Expressed as percentage of waking hours, children with bronchiectasis, on average, were sedentary for 57.5% of the time, in LPA 35.8% of the time, and in MVPA 6.7% of the time. Boys consistently exhibited higher levels of physical activity and less sedentary time than girls; however, none of the sex differences were statistically significant at the 0.05 level.

**Table 2:** Means and 95% confidence intervals for physical activity and sedentary behaviors

	Total	Boys	Girls
Sedentary (min/day)	418.7	410.3	427.1
	(399.5–437.9)	(386.3–434.4)	(396.9–457.3)
Light PA (min/day)	260.9	266.2	255.7
	(247.2–274.7)	(249.0–283.4)	(234.1–277.3)
MVPA (min/day)	48.6	51.7	45.4
	(40.4–56.7)	(41.5–61.9)	(32.6–58.3)
Steps (per day)	8229.5	8422.1	8036.9
	(7458.8–9000.2)	(7458.0–9386.2)	(6825.9–9247.8)

*PA* physical activity, *MVPA* the sum of moderate to vigorous physical activity

Table 3 displays means and 95% CI’s for the weekday and weekend physical activity variables adjusted for age and accelerometer wear time. Participants exhibited higher levels of physical activity and less sedentary time on weekdays compared to weekend days; however, none of the differences were statistically significant. A similar pattern of findings was observed when weekday and weekend physical activity and sedentary behaviours were examined by sex.

**Table 3:** Means and 95% confidence intervals for physical activity and sedentary behaviors on weekday vs weekend days

	Total		Boys		Girls	
	Weekdays	Weekends	Weekdays	Weekends	Weekdays	Weekends
Sedentary (min/day)	417.6	421.1	405.1	431.1	430.1	411.1
	(389.2–445.9)	(381.9–460.3)	(369.6–440.6)	(382.1–480.1)	(385.5–474.7)	(349.5–472.6)
Light PA (min/day)	266.4	247.5	270.6	253.8	262.2	241.2
	(253.4–279.5)	(224.4–270.6)	(254.4–286.9)	(224.9–282.6)	(241.8–282.7)	(205.0–277.5)
MVPA (min/day)	52.2	38.9	54.7	41.4	49.7	36.3
	(43.1–61.3)	(30.1–47.6)	(43.2–66.1)	(30.5–52.4)	(35.4–64.1)	(22.5–50.1)
Steps (per day)	8682.4	6985.9	8885.9	6952.4	8479.0	7019.4
	(7893.2–9471.6)	(5956.3–8015.4)	(7898.7–9873.1)	(5664.5–8240.3)	(7239.0–9718.9)	(5401.82–8637.0)

*PA* physical activity, *MVPA* sum of moderate and vigorous physical activity

**Fig. 1:**
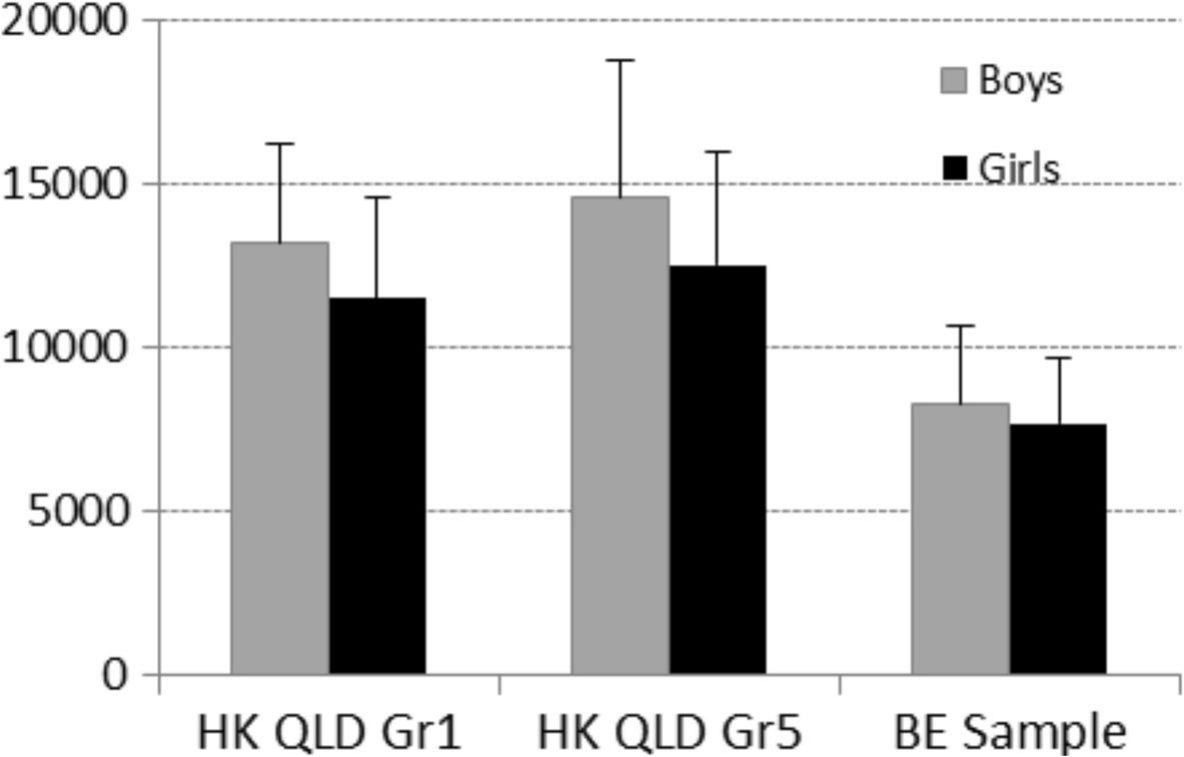
Mean daily steps of children with bronchiectasis compared to healthy controls from the Healthy Kids Queensland Survey. (BE = bronchiectasis, HK QLD Gr1 = Healthy Kids Queensland Survey – 1st Grade Students, HK QLD Gr5 = Healthy Kids Queensland Survey – 5th Grade students)

Overall, 5.6% (*n* = 2) of the participants met the 60-min daily MVPA recommendation. In comparison, 42.1% of healthy children participating in the Queensland Preventive Health Survey met the daily MVPA recommendation. Figure 1 illustrates the mean daily step counts of children with bronchiectasis and normative data from the Healthy Queensland Kids Survey [31]. On average, children with bronchiectasis accumulated 8229 ± SD 2360 steps/day (boys: 8422 ± SD 473, girls: 8037 ± 594), well below the recommended 12,000 steps/day. In comparison, daily step counts in healthy children ranged from 11,500–14,500 steps/day.

## Discussion

Using objective measuring tools, we found that the vast majority of our sample of children with bronchiectasis was insufficiently active for health benefit. Less than 6% of children achieved the recommended daily 60 min of MVPA. In contrast, 42% of healthy children in the normative comparison group met the daily 60 min guideline. On average, children with bronchiectasis accumulated 48 min of MVPA daily, with boys displaying marginally higher levels of MVPA than girls. Collectively, these findings highlight the need for effective and sustainable programs to promote regular physical activity in children with bronchiectasis.

Our findings are similar with those of Aznar et al. [18] who objectively measured the physical activity levels of 47 children and adolescents with CF. Applying the same monitoring protocol and data reduction procedures used in the current study, only one participant in their study (2.1%) met the daily 60 min MVPA guideline. In comparison, 34.2% of healthy age and gender-matched controls met the guideline. More recently, Mackintosh et al. [33] used the ActiGraph GT3X accelerometer to objectively measure physical activity in 36 children and adolescents with CF. In contrast with the results of the current study and those of Aznar et al. [18], 38.9% of children with CF met the daily 60 min MVPA guideline. The discrepancy in findings may be attributable, at least in part, to differences in the accelerometer data reduction protocol. Mackintosh et al. [33] converted the accelerometer data into daily time spent in MVPA by applying the age-specific cut points of Freedson et al. [34] which have been shown to significantly overestimate MVPA levels, particularly among younger children [28]. Furthermore, children were classified as meeting the guideline if they averaged 60 min of MVPA rather than accumulating 60 min of MVPA on each day, as specified in the recommendation. Such findings highlight the need for a standardized approach for measuring physical activity objectively in children with chronic respiratory disease. This would make it possible to directly compare findings of studies and draw valid conclusions regarding physical activity levels in this population.

In the light of emerging evidence identifying prolonged sitting as an independent risk factor for increased cardio metabolic risk in children [14], daily time spent in sedentary behavior has become a topic of considerable public health importance. In the current study, children with bronchiectasis spent more than half of their waking hours being sedentary, with girls accumulating more sedentary time than boys. Similar results were reported by Aznar et al. [18] and Mackintosh et al. [33]. These findings suggest that children with bronchiectasis could improve their health and well-being by replacing sedentary time with light- or moderate-intensity physical activities. Previous studies have shown that encouraging a TV turn off week and the use of standing desks might be effective strategies to reduce prolonged sitting in healthy children [35]. The extent to which such interventions are effective in children with chronic respiratory disease is therefore an important topic for future research.

The current study had several limitations which should be considered. First, participants were recruited from a single public hospital in Brisbane, Australia and cannot be considered representative of all children with bronchiectasis. In addition, because patients with less than 4 valid monitoring days (including 1 weekend day) were excluded from the analysis, we cannot rule out the possibility of selection bias (i.e., more active patients were more likely to be included in the analytic sample than low-active patients). However, given that only 2 participants in our sample met the 60 min MVPA guideline, this is unlikely. Future studies should evaluate physical activity levels in larger, more representative samples of children with bronchiectasis. Samples should be sufficiently large and diverse to determine how physical activity levels vary by demographic, socioeconomic status and health characteristics. The assessment of physical activity in indigenous children with bronchiectasis is also a priority for future research. Second, no healthy child control group was included. However, normative data from two population-representative health surveys was used to compare physical activity levels and daily step counts to healthy children. This showed that the children from the current sample were substantially less active than healthy children and that fewer children with bronchiectasis meet physical activity guidelines. Importantly, because estimates from one survey were based on self-reported physical activity and prone to recall and social desirability bias, this comparison should be interpreted with caution. However, it should be noted that objectively measured step counts in the current study and the Healthy Queensland Kids Survey were directly comparable and confirmed that bronchiectasis patients in our study exhibited lower levels of physical activity than their healthy counterparts. In support of our comparison with normative data, the percentage of children meeting the 60 min MVPA guideline in the Queensland Preventive Health Survey based on self-report data was comparable to estimates reported in healthy Australian children using the ActiGraph accelerometer to objectively measure physical activity. Roman-Vinas et al. [36] assessed compliance with the guidelines in 12 different countries, including a sample of 451 Australian children aged 9 to 11 years. Applying the same accelerometer count cut-point for MVPA used in the current study, just over 55% of the Australian sample met the daily 60 min MVPA guideline. Across all 12 countries, 44.1% of children met the daily physical activity guideline. A third limitation was that the accelerometer count cut-points used to determine time spent in MVPA were developed on healthy children. Nevertheless, they were shown to be the most accurate in a large sample of healthy children [28] and were used in a previous study involving children with CF [18]. Fourth, although accelerometers provide valid estimates of the frequency, intensity, and duration of physical activity, they do not provide information about the type of activities performed [37]. Fifth and finally, the limited sample size did not allow us to evaluate potentially important age-related differences in physical activity levels, examine activity differences related to disease severity, medication use, and socioeconomic status, or explore reasons for their lack of physical activity. A decline of physical activity over time is typical for healthy children; nevertheless, it would be of interest to determine if this decline starts earlier in children with chronic respiratory disease.

## Conclusion

Using an accelerometer to objectively measure physical activity, we found generally low MVPA levels in 36 children with bronchiectasis. Only 2 children (5.6%) achieved the recommended 60 min of daily MVPA. When examined as a percentage of waking hours, they spent more than 50% of their time being sedentary. These findings highlight the need for programs to promote physical activity and reduce sedentary time in this patient group. Such programs should be developmentally appropriate and tailored to the needs of children with chronic respiratory disease. Clinicians should be encouraged to monitor and promote physical activity in their patients, and when indicated, refer them to developmentally appropriate therapeutic exercise programs and/or community-based physical activity programs.

## Abbreviations

CF: Cystic fibrosis
LPA: Light-intensity physical activity
MPA: Moderate-intensity physical activity
MVPA: Moderate- to vigorous-intensity physical activity
SED: Sedentary
VPA: Vigorous-intensity physical activity
